# Prognostic significance of AMP-activated protein kinase expression and modifying effect of MAPK3/1 in colorectal cancer

**DOI:** 10.1038/sj.bjc.6605846

**Published:** 2010-08-31

**Authors:** Y Baba, K Nosho, K Shima, J A Meyerhardt, A T Chan, J A Engelman, L C Cantley, M Loda, E Giovannucci, C S Fuchs, S Ogino

**Affiliations:** 1 Department of Medical Oncology, Dana-Farber Cancer Institute and Harvard Medical School, 44 Binney Street, Room JF-215C, Boston, MA 02115, USA; 2 Gastrointestinal Unit, Massachusetts General Hospital and Harvard Medical School, Boston, MA 02114, USA; 3 Cancer Center, Massachusetts General Hospital and Harvard Medical School, Boston, MA 02114, USA; 4 Department of Systems Biology, Beth Israel Deaconess Medical Center and Harvard Medical School, Boston, MA 02115, USA; 5 Department of Pathology, Brigham and Women's Hospital, Boston and Harvard Medical School, Boston, MA 02115, USA; 6 Department of Medicine, Channing Laboratory, Brigham and Women's Hospital and Harvard Medical School, Boston, MA 02115, USA; 7 Departments of Epidemiology and Nutrition, Harvard School of Public Health, Boston, MA 02115, USA

**Keywords:** colon cancer, AMPK, ERK, energy balance, prognosis

## Abstract

**Background:**

AMP-activated protein kinase (AMPK, PRKA) has central roles in cellular metabolic sensing and energy balance homeostasis, and interacts with various pathways (e.g., TP53 (p53), FASN, MTOR and MAPK3/1 (ERK)). AMP-activated protein kinase activation is cytotoxic to cancer cells, supporting AMPK as a tumour suppressor and a potential therapeutic target. However, no study has examined its prognostic role in colorectal cancers.

**Methods:**

Among 718 colon and rectal cancers, phosphorylated AMPK (p-AMPK) and p-MAPK3/1 expression was detected in 409 and 202 tumours, respectively, by immunohistochemistry. Cox proportional hazards model was used to compute mortality hazard ratio (HR), adjusting for clinical and tumoral features, including microsatellite instability, CpG island methylator phenotype, LINE-1 methylation, and *KRAS, BRAF* and *PIK3CA* mutations.

**Results:**

Phosphorylated AMPK expression was not associated with survival among all patients. Notably, prognostic effect of p-AMPK significantly differed by p-MAPK3/1 status (*P*_interaction_=0.0017). Phosphorylated AMPK expression was associated with superior colorectal cancer-specific survival (adjusted HR 0.42; 95% confidence interval (CI), 0.24–0.74) among p-MAPK3/1-positive cases, but not among p-MAPK3/1-negative cases (adjusted HR 1.22; 95% CI: 0.85–1.75).

**Conclusion:**

Phosphorylated AMPK expression in colorectal cancer is associated with superior prognosis among p-MAPK3/1-positive cases, but not among p-MAPK3/1-negative cases, suggesting a possible interaction between the AMPK and MAPK pathways influencing tumour behaviour.

Colorectal cancer is the fourth most common malignancy and the second most frequent cause of cancer-related death in the United States, with approximately 50 000 cancer-related deaths in 2009 (Jemal *et al*, 2009). Colorectal cancer arises through a multistep carcinogenic process in which genetic and epigenetic alterations (e.g., microsatellite instability (MSI), CpG island methylation, mutations in *KRAS*, *BRAF* and *PIK3CA*) accumulate in a sequential manner. A better understanding of molecular alterations in colorectal cancer may be of great clinical importance. *KRAS* mutational status of stage IV colorectal cancer is a predictive biomarker for anti-EGFR treatment (Loupakis *et al*, 2009). In addition, *BRAF* mutation identifies a subgroup of patients with unfavourable prognosis (Ogino *et al*, 2009; Roth *et al*, 2010).

AMP-activated protein kinase (AMPK; PRKA, the HUGO-approved official gene stem symbol) is a heterotrimeric serine/threonine protein kinase, which acts as a cellular sensor for energy balance status. AMP-activated protein kinase is phosphorylated by its upstream kinase STK11 (LKB1) in response to an increase in cellular AMP/ATP ratio (Shackelford and Shaw, 2009). It regulates cell proliferation and growth by inhibition of the MTOR pathway and fatty acid synthesis, and activation of the TP53-CDKN1A (p21) pathway (Figure 1) (Inoki and Guan, 2009). The MAPK3/1 (extracellular signal-regulated kinase (ERK)1/2) pathway is activated by extracellular and intracellular mitogenic stimuli and has crucial roles in cellular differentiation, proliferation and survival (Schubbert *et al*, 2007). Interactions between the STK11 (LKB1)-AMPK pathway and the MAPK3/1 pathway in human cancer cells including colon cancer cells have been documented (Esteve-Puig *et al*, 2009; Zheng *et al*, 2009; Kim *et al*, 2010). AMP-activated protein kinase activation is cytotoxic to various cancer cell types, and inhibits tumour growth (Buzzai *et al*, 2007; Zakikhani *et al*, 2008), supporting AMPK as a tumour suppressor and a potential target for cancer therapy and chemoprevention (Fay *et al*, 2009). Thus, better understanding of the mechanism and consequence of AMPK activation in human cancer is increasingly important. To our best knowledge, no previous study has examined AMPK status and patient prognosis in human colorectal cancer. Given potential roles of AMPK as a regulator of cellular metabolism and a tumour suppressor related to cellular signaling pathways (e.g., the MAPK3/1 pathway), we hypothesised that AMPK might interact with MAPK3/1 to modify tumour behaviour.

To test this hypothesis, we utilised a database of 718 stage I–IV colorectal cancers in two prospective cohort studies, and examined the prognostic role of phosphorylated-AMPK expression and modifying effect of MAPK3/1. As a result of our database with other tumoral variables including FASN, TP53, *KRAS*, *BRAF* and *PIK3CA* mutations, MSI, the CpG island methylator phenotype (CIMP) and LINE-1 methylation, we could examine the relationship between AMPK status and other molecular features, as well as interactive prognostic effect of AMPK and other molecular events.

## Materials and methods

### Study group

We utilised the databases of two independent, prospective cohort studies; the Nurses’ Health Study (*N*=121 701 women followed since 1976), and the Health Professionals Follow-Up Study (*N*=51 529 men followed since 1986) (Chan *et al*, 2007). A subset of the cohort participants developed colorectal cancers during prospective follow-up. We collected paraffin-embedded tissue blocks from hospitals where patients underwent tumour resections. We excluded cases for which preoperative treatment was administered. Tissue sections from all colorectal cancer cases were reviewed by a pathologist (SO) unaware of other data. The tumour grade was categorised as low *vs* high (⩾50 *vs* <50% gland formation). The type of tumour border (expansile or infiltrative) was categorised as previously published criteria (Ogino *et al*, 2006e). On the basis of the availability of adequate tissue specimens and follow-up data, a total of 718 colorectal cancers (diagnosed up to 2004) were included. Patients were observed until death or 30 June 2008, whichever came first. Among our cohort studies, there was no significant difference in demographic features between cases with tissue available and those without available tissue (Chan *et al*, 2007). This current analysis represents a new analysis of p-AMPK and p-MAPK3/1 on the existing colorectal cancer database that has been previously characterised for CIMP, MSI, *KRAS, BRAF, PIK3CA*, LINE-1 methylation and clinical outcome (Ogino *et al*, 2007, 2008b, 2009). However, in any of our previous studies, we have neither examined AMPK or MAPK3/1 expression. Informed consent was obtained from all study subjects. Tissue collection and analyses were approved by the Harvard School of Public Health and Brigham and Women's Hospital Institutional Review Boards.

### Sequencing of *KRAS*, *BRAF* and *PIK3CA* and MSI analysis

DNA was extracted from tumour tissue, and PCR and pyrosequencing targeted for *KRAS* (codons 12 and 13) (Ogino *et al*, 2005), *BRAF* (codon 600) (Ogino *et al*, 2006d) and *PIK3CA* (exons 9 and 20) were performed (Nosho *et al*, 2008b). The status of MSI was determined by analysing variability in the length of the microsatellite markers from tumour DNA compared with normal DNA. We used D2S123, D5S346, D17S250, BAT25, BAT26 BAT40, D18S55, D18S56, D18S67 and D18S487 (Ogino *et al*, 2006a). Microsatellite instability-high was defined as the presence of instability in ⩾30% of the markers, and MSI-low/microsatellite stability (MSS) as instability in 0–29% of the markers according to the Bethesda guideline (Boland *et al*, 1998).

### Methylation analyses for CpG islands and LINE-1

Using validated bisulphite DNA treatment and real-time PCR (MethyLight), we quantified DNA methylation in eight CIMP-specific promoters (*CACNA1G, CDKN2A* (p16), *CRABP1, IGF2, MLH1, NEUROG1, RUNX3* and *SOCS1*) (Weisenberger *et al*, 2006; Ogino *et al*, 2006c, 2007). The CIMP-high was defined as the presence of ⩾6 out of 8 methylated promoters, CIMP-low/0 as 0 out of 8–5 out of 8 methylated promoters, based on a distribution of tumours and *BRAF* and *KRAS* mutation frequencies (Ogino *et al*, 2007). Concordance between our eight-marker panel and the Weisenberger panel (Weisenberger *et al*, 2006) was very high (99%, *κ*=0.94, *P*<0.0001) (Nosho *et al*, 2008a). In order to accurately quantify relatively high methylation levels in LINE-1, we utilised pyrosequencing (Ogino *et al*, 2008a; Irahara *et al*, 2010).

### Immunohistochemistry

**Figure 2:**
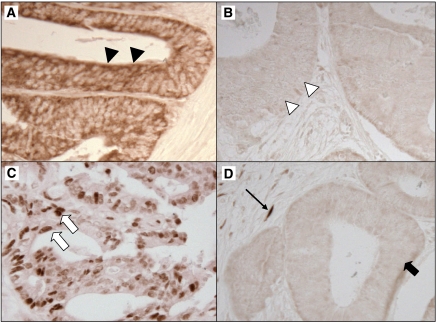
Phosphorylated AMPK and p-MAPK3/1 expression in colorectal cancer. (**A**) Positive for p-AMPK cytoplasmic expression (arrowheads). (**B**) Negative for p-AMPK expression (white arrowheads). (**C**) Positive for p-MAPK3/1 nuclear expression (white arrows). (**D**) Negative for p-MAPK3/1 expression (block arrow). Stromal cells serve as an internal positive control for p-MAPK3/1 expression (arrow).

Tissue microarrays were constructed as previously described (Ogino *et al*, 2006b). Methods of immunohistochemistry were previously described for TP53 and FASN (fatty acid synthase) (Ogino *et al*, 2006a, 2008c). For AMP-activated protein kinase (AMPK, PRKA), we evaluated PRKAA (AMPK*α*) Thr172 phosphorylation status (Figure 2). Deparaffinised tissue sections in Antigen Retrieval Citra Solution (Biogenex Laboratories, San Ramon, CA, USA) were treated with microwave in a pressure cooker (25 min). Tissue sections were incubated with 5% normal goat serum (Vector Laboratories, Burlingame, CA, USA) in phosphate-buffered saline (30 min). Primary antibody against p-AMPK (rabbit monoclonal anti-phospho-AMPK*α* (Thr172) (40H9), 1 : 100 dilution; Cell Signaling Technology, Boston, MA, USA) was applied (Ji *et al*, 2007; Contreras *et al*, 2008; Hadad *et al*, 2009; Vazquez-Martin *et al*, 2009; Zheng *et al*, 2009), and the slides were maintained at 4 °C for overnight, followed by rabbit secondary antibody (Vector Laboratories) (60 min), an avidin–biotin complex conjugate (Vector Laboratories) (60 min), diaminobenzidine (5 min) and methyl-green counterstain. Cytoplasmic p-AMPK expression was recorded as no expression, weak expression or moderate/strong expression with the percentage of positive tumour cells. The CIMP status reflects global epigenomic aberrations in tumour cells (Ogino and Goel, 2008) and may influence energy sensing status of cancer cells. Indeed, epidemiological studies has suggested a potential link between CIMP and energy metabolism in colorectal cancer; high intake of high-fat dairy products is associated with CIMP-high rectal cancers (Slattery *et al*, 2010) and exposure to a period of severe transient energy restriction during adolescence is inversely associated with the risk of having a CIMP-high tumour later in life (Hughes *et al*, 2009). In addition, the relationship between CIMP and a molecular alteration related to energy metabolism (e.g., SIRT1) has been reported (Nosho *et al*, 2009). Thus, we explored the use of CIMP status to determine a cutoff for p-AMPK positivity; there was no alternative biologically based method in our cohort studies. First, we categorised tumours according to intensity of p-AMPK and the fraction of p-AMPK-expressing cells. In our initial exploratory analysis, we randomly selected 358 tumours as a training set, leaving the remaining 360 tumours as a validation set. Using the training set, the frequency of CIMP-high in each category was: 25% (29 out of 117) in tumours with no expression; 21% (11 out of 53) in tumours with weak expression in 1–19% of tumour cells; 12% (14 out of 117) in tumours with weak expression in 20–100% of cells; 7.8% (5 out of 64) in tumours with moderate or strong expression. Thus, p-AMPK positivity was defined as the presence of weak cytoplasmic expression in ⩾20% of tumour cells or moderate/strong expression in any fraction of tumour cells. In the remaining validation set, p-AMPK expression defined by the training set was inversely associated with CIMP-high (odds ratio (OR) 0.45; 95% confidence interval (CI): 0.25–0.83; *P*=0.0094), validating the cutoff although it might not be the most biologically reasonable cutoff. In addition, to evaluate whether p-AMPK expressions in tumour centre and invasive front were different, we stained 20 whole tissue sections for p-AMPK and recorded p-AMPK expression status of both tumour centre and tumour invasive front.

For phosphorylated-MAPK3/1 (p-MAPK3/1), the same protocol with p-AMPK was used except for primary antibody (rabbit monoclonal anti-phospho-p44/42 MAPK (ERK1/2) (Thr202/Thr204) (20G11), 1 : 100 dilution; Cell Signaling Technology). Nuclear p-MAPK3/1 expression was recorded as no, weak, moderate or strong expression with the percentage of positive tumour cells. Considering that MAPK3/1 is downstream of the RAF pathway, we used *BRAF* mutation frequency to determine a cutoff for p-MAPK3/1 positivity. First, we categorised tumours according to the intensity of p-MAPK3/1 expression. Using the training set, the frequency of *BRAF* mutation in each category was: 17% (35 out of 206) in tumours with no expression; 8.5% (6 out of 71) in tumours with weak expression; 7.0% (4 out of 57) in tumours with moderate or strong expression. Thus, p-MAPK3/1 positivity was defined as weak/moderate/strong expression. In the remaining validation set, p-MAPK3/1 expression defined by the training set was inversely associated with *BRAF* mutation (OR 0.42; 95% CI: 0.20–0.90; *P*=0.023), validating the cutoff although it might not be the most biologically reasonable cutoff.

Appropriate positive and negative controls were included in each run of immunohistochemistry. Each immunohistochemical maker was interpreted by one of the investigators (p-AMPK and p-MAPK3/1 by YB; TP53 and FASN by SO) unaware of other data. For agreement studies, a random selection of 108–246 cases was examined for each marker by a second observer (by KN) unaware of other data. The concordance between the two observers (all *P*<0.0001) was 0.82 (*κ*=0.63; *N*=137) for p-AMPK, 0.86 (*κ*=0.70; *N*=137) for p-MAPK3/1, 0.87 (*κ*=0.75; N=108) for TP53 and 0.93 (*κ*=0.57; *N*=246) for FASN, indicating good-to-substantial agreement.

### Statistical analysis

For all statistical analyses, we used SAS program (Version 9.1, SAS Institute, Cary, NC, USA). All *P-*values were two-sided, and statistical significance was set at *P*=0.05. Nonetheless, when we performed multiple hypothesis testing, a *P*-value for significance was adjusted by Bonferroni correction to *P*=0.0029 (=0.05/17). For categorical data, the *χ*^2^ test was performed. For survival analysis, Kaplan–Meier method and log-rank test was used. For analyses of colorectal cancer-specific mortality, deaths as a result of causes other than colorectal cancer were censored. To assess independent effect of p-AMPK on mortality, tumour stage (I, IIA, IIB, IIIA, IIIB, IIIC, IV, unknown) was used as a stratifying variable in Cox models using the ‘strata’ option in the SAS ‘proc phreg’ command to avoid residual confounding and overfitting. We constructed a multivariate, stage-stratified Cox proportional hazards model to compute a hazard ratio (HR) according to p-AMPK status, initially including sex, age at diagnosis (continuous), body mass index (BMI, <30 *vs* ⩾30 kg m^–2^), family history of colorectal cancer in any first-degree relative (present *vs* absent), tumour location (rectum *vs* colon), tumour grade (low *vs* high), tumour border (infiltrative *vs* expansile), CIMP (high *vs* low/0), MSI (high *vs* low/MSS), LINE-1 methylation (continuous), *BRAF*, *KRAS*, *PIK3CA*, TP53 and FASN. A backward stepwise elimination with a threshold of *P*=0.20 was used to select variables in the final model. For cases with missing information in any of categorical variables (tumour location (1.2%), MSI (1.9%), *BRAF* (1.7%), *KRAS* (1.3%), *PIK3CA* (10%), TP53 (0.6%) and FASN (1.0%)), we included those cases in a majority category of a given covariate to avoid overfitting. We confirmed that excluding cases with missing information in any of the covariates did not substantially alter results (data not shown). The proportionality of hazard assumption was satisfied by evaluating time-dependent variables, which were the cross-product of the AMPK variable and survival time (*P*>0.05). An interaction was assessed by including the cross product of p-AMPK variable and another variable of interest (without data-missing cases) in a multivariate Cox model, and the Wald test was performed. Backward stepwise elimination with a threshold of *P*=0.20 was used to select variables in the final model. A *P*-value for significance was adjusted to *P*=0.0029 by Bonferroni correction for multiple hypothesis testing.

## Results

### AMPK expression in colorectal cancer

To evaluate whether phosphorylated AMPK (p-AMPK, p-PRKA) expressions in tumour centre and invasive front were different, we stained 20 whole tissue sections for p-AMPK and recorded p-AMPK expression status of both tumour centre and tumour invasive front. In 16 of 20 sections, tumour centre and tumour invasive front showed concordant expression status, indicating that p-AMPK expressions in tumour centre and invasive front were not different in most cases. Furthermore, whole tissue section-based expression status and TMA-based expression status were concordant in 18 of 20 cases, indicating that expression status determined using TMA represented expression status of tumour as a whole in a vast majority of cases.

Among 718 colorectal cancers in the two prospective cohort studies, we detected p-AMPK in 409 tumours (57%) by immunohistochemistry. Phosphorylated AMPK expression was associated with p-MAPK3/1 expression (*P*<0.0001) and inversely with high tumour grade (*P*=0.0009), MSI-high (*P*=0.0021) and CIMP-high (*P*<0.0001) (Table 1).

### AMPK expression and prognosis in colorectal cancer

**Figure 3:**
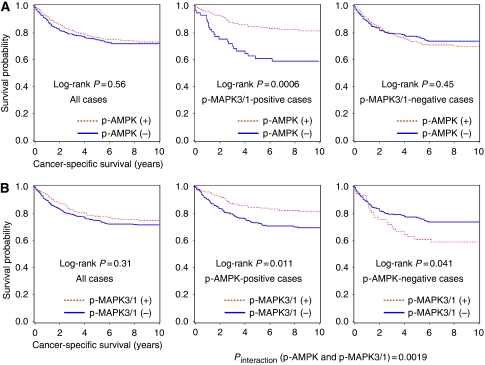
Kaplan–Meier curves for colorectal cancer-specific survival. (**A**) p-AMPK status and survival of colorectal cancer patients. The left panel includes all eligible cases, the middle panel includes p-MAPK3/1-positive cases, and the right panel includes p-MAPK3/1-negative cases. (**B**) p-MAPK3/1 status and survival of colorectal cancer patients. The left panel includes all eligible cases, the middle panel includes p-AMPK-positive cases, and the right panel includes p-AMPK-negative cases.

Among the 718 patients (with median follow-up of 129 months for censored patients), there were 306 deaths, including 194 colorectal cancer-specific deaths. In Kaplan–Meier or Cox regression analysis, p-AMPK status was not significantly associated with colorectal cancer-specific or overall survival among all eligible patients (Figure 3A, Table 2).

### Modifying effect of p-MAPK3/1 expression on p-AMPK expression in survival analysis

Considering experimental data on the interaction between AMPK and MAPK3/1 (Esteve-Puig *et al*, 2009; Zheng *et al*, 2009; Kim *et al*, 2010), we assessed whether p-MAPK3/1 status could modify the prognostic effect of p-AMPK expression. We found a significant modifying effect of p-MAPK3/1 expression on the relation between p-AMPK expression and mortality (*P*_interaction_=0.0017 (for colorectal cancer-specific mortality) and *P*_interaction_=0.0026 (for overall mortality)). Among patients with p-MAPK3/1-positive tumour, p-AMPK expression was associated with a significant decrease in colorectal cancer-specific mortality (adjusted HR 0.42; 95% CI: 0.24–0.74), whereas p-AMPK expression was not significantly related with prognosis among patients with p-MAPK3/1-negative tumour (adjusted HR 1.22; 95% CI: 0.85–1.75; p-AMPK-positive *vs* negative) (Table 3).

In Kaplan–Meier method, the differential prognostic effect of p-AMPK expression according to p-MAPK3/1 expression status was evident (Figure 3A). Phosphorylated AMPK expression was associated with longer colorectal cancer-specific survival (log-rank *P*=0.0006) among p-MAPK3/1-positive cases, whereas p-AMPK expression was not significantly associated with survival among p-MAPK3/1-negative cases (log-rank *P*=0.45).

### Prognostic effect of p-MAPK3/1 expression in strata of p-AMPK status

In Kaplan–Meier analysis, p-MAPK3/1 was not significantly associated with colorectal cancer-specific survival (log-rank *P*=0.31) (Figure 3B) or overall survival (log-rank *P*=0.68). In light of the significant interaction between p-AMPK and p-MAPK3/1 (*P*_interaction_=0.0017), we examined the prognostic effect of p-MAPK3/1 expression in strata of p-AMPK expression status. Among p-AMPK-negative cases, p-MAPK3/1 expression was significantly associated with inferior colorectal cancer-specific survival (adjusted HR 1.94; 95% CI: 1.17–3.24; p-MAPK3/1-positive *vs* negative tumours). In contrast, among p-AMPK-positive cases, p-MAPK3/1 expression was significantly associated with superior colorectal cancer-specific survival (adjusted HR 0.55; 95% CI: 0.35–0.86) (Table 3). A similar interaction was observed in overall mortality analysis (*P*_interaction_=0.0026).

### Stratified analysis of p-AMPK expression and mortality

We examined whether the influence of p-AMPK expression on colorectal cancer-specific survival was modified by any of the other variables including sex, age, BMI, family history of colorectal cancer, tumour location, stage, tumour grade, CIMP, MSI, *BRAF*, *KRAS*, *PIK3CA*, LINE-1 methylation, TP53 and FASN. We did not observe a significant modifying effect by any of the variables (all *P*_interaction_>0.10). Notably, there was no significant interaction between p-AMPK and mutation in *KRAS* or *BRAF* (*P*_interaction_=0.12 for *BRAF* and *P*_interaction_=0.30 for *KRAS*).

## Discussion

We conducted this study to examine prognostic significance of p-AMPK (phosphorylated AMP-activated protein kinase; p-PRKA) expression in a large cohort of colorectal cancers. To our best knowledge, no previous study has examined its prognostic role in human colorectal cancer. Considering a pivotal role of AMPK as a regulator of cellular metabolism and the relationship of AMPK with the MAPK3/1 (ERK1/2) pathway and other signaling pathways, we hypothesised that cellular AMPK might interact with MAPK3/1 to modify tumour behaviour. Notably, we found that the prognostic effect of p-AMPK expression differed according to p-MAPK3/1 status. Phosphorylated AMPK expression was associated with superior survival among p-MAPK3/1-positive cases, but not among p-MAPK3/1-negative cases. Our results support an interaction between the AMPK and MAPK3/1 pathways in colorectal cancer cells to modify tumour behaviour.

Examining molecular changes or prognostic factors is important in cancer research (Fluge *et al*, 2009; Gaber *et al*, 2009; Jubb *et al*, 2009; Rasheed *et al*, 2009; Kontos *et al*, 2010; Rego *et al*, 2010; Zlobec *et al*, 2010). Accumulating evidence suggests that AMPK acts as a tumour suppressor. STK11 (LKB1) has been identified as an upstream activator of AMPK (Shackelford and Shaw, 2009), and TSC2, which is a negative regulator of MTOR, is a downstream effector of AMPK (Inoki and Guan, 2009). Experimental studies have shown that AMPK activation inhibits cancer cell proliferation and growth (Buzzai *et al*, 2007; Zakikhani *et al*, 2008). In a study using 354 breast cancers (Hadad *et al*, 2009), p-AMPK expression was not significantly associated with prognosis, but modifying effect of MAPK3/1 was not examined. To our knowledge, no previous study has examined the prognostic role of AMPK in colorectal cancer.

Considering experimental data on the link between the STK11 (LKB1)-AMPK and MAPK3/1 pathways, the modifying effect of MAPK3/1 on AMPK may not be surprising. In colon cancer cells, AMPK potentially inhibits the MAPK3/1 pathway; inhibition of AMPK by expressing a dominant-negative form potentiates MAPK3/1 activation under glucose deprivation (Kim *et al*, 2010). Selenium, an essential trace element, blocks the carcinogenic agent-induced MAPK3/1 activation via AMPK (Hwang *et al*, 2006). AMP-activated protein kinase is rapidly activated by cisplatin and suppresses an apoptotic signal via MAPK3/1 in colon cancer cells (Kim *et al*, 2008). A study using melanoma cells (Zheng *et al*, 2009) has shown that the MAPK3/1 pathway phosphorylates STK11 on Ser325 and Ser428 and promotes the uncoupling of AMPK from STK11, which negatively regulates AMPK. Regulation of AMPK activity by the MAPK3/1 pathway, independent of STK11 Ser428 phosphorylation, has also been reported (Esteve-Puig *et al*, 2009). In fibroblast cells, AMPK differentially inhibits the MAPK3/1 pathway by inhibiting RAS activation or stimulating the RAS-independent pathway in response to cellular energy status (Kim *et al*, 2001). We should also consider the complex TSC2-MTOR axis-mediated linkage. AMP-activated protein kinase suppresses MTOR activity directly by phosphorylating MTOR at Thr2446 and indirectly by phosphorylating TSC2 at Thr1227 and Ser1345 and increasing the activity of TSC-complex (Inoki and Guan, 2009). MAPK3/1 increases MTOR activity by phosphorylating TSC2 at Ser540 and Ser664, which causes the attenuation of TSC2 (Ma *et al*, 2005). Our findings may support the hypothesis that AMPK activation can make a strong impact on tumour behaviour as the ‘brake’ only when MAPK3/1 is active. Additional studies are needed to confirm our findings and elucidate the exact mechanism of effect of MAPK3/1 on AMPK to modify tumour behaviour.

Our study has shown that MAPK3/1 activation has a differential effect on patient mortality according to AMPK status; p-MAPK3/1 expression is associated with good prognosis among p-AMPK-positive patients, but with poor prognosis among p-AMPK-negative patients. It remains controversial how MAPK3/1 activation affects behaviour of different cancers (Milde-Langosch *et al*, 2005; Pelloski *et al*, 2006). A study on 135 colorectal cancers has shown that p-MAPK3/1 expression is associated with poor prognosis (Schmitz *et al*, 2007). In contrast to that study (*N*=135), our study evaluated the expression status of both p-MAPK3/1 and p-AMPK in a much larger cohort of 718 colorectal cancers. In addition, we assessed the interactive effect of p-MAPK3/1 and p-AMPK expression independent of other molecular events that have been documented to be critical in colorectal carcinogenesis.

Recently, AMPK has been proposed as a potential target for cancer prevention and treatment, and various AMPK activators have been preclinically assessed (Fay *et al*, 2009). Among them, metformin, a widely used anti-diabetic drug, has shown promising results (Buzzai *et al*, 2007; Zakikhani *et al*, 2008). Metformin may have two properties of potential oncologic relevance: it has a direct, STK11-AMPK pathway-dependent growth inhibitory effect and decreases systemic insulin levels (Pollak, 2008). Interestingly, two observational studies have shown that diabetic patients treated with metformin experienced a lower incidence of any kind of cancer and a lower cancer-related mortality (Evans *et al*, 2005; Bowker *et al*, 2006). Hereafter, in clinical trial of this drug, examining AMPK status in cancer tissue might be important. In this regard, our findings may have clinical implications. In addition, drugs targeting the MAPK3/1 pathway are intensively being developed and tested in clinical trials for various human cancers (Beeram *et al*, 2005). Although the usefulness of MAPK3/1 expression as a biomarker for sensitivity to these drugs is uncertain (Yeh *et al*, 2009), further understanding of the linkage between the AMPK and MAPK3/1 pathways could potentially provide useful information for refinement of therapeutic strategies.

We found significant relations of p-AMPK expression with MSI-high and CIMP-high. MSI and CIMP status reflect global genomic and epigenomic aberrations in tumour cells, and hence, are associated with various clinical, pathologic and molecular features (Ogino and Goel, 2008). Considering the known relationship between MSI and/or CIMP and molecular alterations related to energy metabolism (Ogino *et al*, 2007b; Nosho *et al*, 2009), MSI and CIMP may influence energy sensing status of cancer cells.

There are limitations in this study. For example, data on cancer treatment were limited. Nonetheless, it is unlikely that chemotherapy use substantially differed according to AMPK status in tumour, because such data were unavailable for treatment decision making. In addition, our multivariate survival analysis finely adjusted for disease stage (I, IIA, IIB, IIIA, IIIB, IIIC, IV, unknown), on which treatment decision making was mostly based. As another limitation, beyond cause of mortality, data on cancer recurrence were unavailable in these cohort studies. Nonetheless, colorectal cancer-specific survival might be a reasonable surrogate of colorectal cancer-specific outcome. Furthermore, the cutoffs for p-AMPK and p-MAPK3/1 used in this current study need to be validated in an independent data set.

There are advantages in utilising the database of the two prospective cohort studies, the Nurses’ Health Study and the Health Professionals Follow-Up Study, to examine prognostic significance of tumour AMPK expression. Anthropometric measurements, family history, cancer staging, and other clinical, pathologic, and tumour molecular data were prospectively collected, blinded to patient outcome. Cohort participants who developed cancer were treated at hospitals throughout the United States, and thus more representative colorectal cancers in the US population than patients in one to several academic hospitals. There was no demographic difference between cases with tumour tissue analysed and those without tumour tissue analysed (Chan *et al*, 2007). Finally, our rich tumour database enabled us to simultaneously assess pathologic and tumoral molecular correlates and control for confounding by a number of tumoral molecular alterations.

In summary, we have shown that AMPK activation is associated with good prognosis among MAPK3/1-activated colorectal cancer patients, while AMPK activation is not associated with prognosis among MAPK3/1-inactive cancer patients. Additional studies are necessary to confirm our observations and to elucidate exact mechanisms by which AMPK and MAPK3/1 interact and affect tumour behaviour. This possible interaction between the AMPK and MAPK3/1 pathways may have considerable implications because both pathways are potential targets for cancer treatment and prevention. In this regard, examining AMPK and MAPK3/1 status in cancer tissue may be important in future clinical trials.

## Figures and Tables

**Figure 1:**
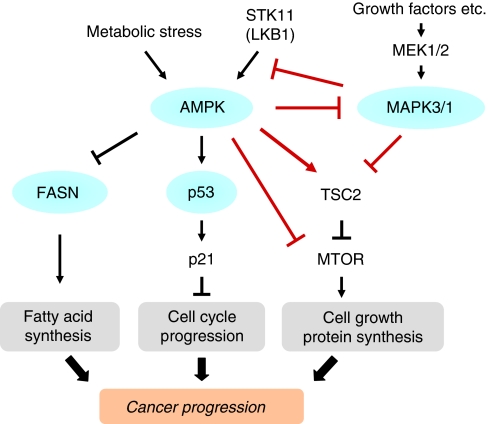
Schematic representation of the AMPK pathway in relation to various molecules. Arrows and lines indicate the pathways potentially related with the complex interaction between AMPK and MAPK3/1. Circles indicate the tissue markers analysed in our current study.

**Table 1:** p-AMPK expression in colorectal cancer, and clinical, pathologic and molecular features

**Clinical, pathologic**		**p-AMPK expression**		
**or molecular feature**	**Total *N***	**Negative**	**Positive**	***P-*value**
All cases	718	309	409	
*Sex*				0.051
Male	259 (36%)	99 (32%)	160 (39%)	
Female	459 (64%)	210 (68%)	249 (61%)	
				
*Age (years)*				
⩽59	143 (20%)	71 (23%)	72 (18%)	0.071
60–69	301 (42%)	116 (38%)	185 (45%)	
⩾70	274 (38%)	122 (39%)	152 (37%)	
*BMI*				0.69
<30 kg m^–2^	594 (83%)	254 (82%)	340 (83%)	
⩾30 kg m^–2^	123 (17%)	55 (18%)	68 (17%)	
				
*Family history of colorectal cancer*				0.66
(−)	554 (77%)	236 (76%)	318 (78%)	
(+)	164 (23%)	73 (24%)	91 (22%)	
				
*Tumour location*				0.61
Proximal colon (cecum to transverse)	347 (49%)	155 (51%)	192 (48%)	
Distal colon (splenic flexure to sigmoid)	220 (31%)	89 (29%)	131 (32%)	
Rectum	140 (20%)	61 (20%)	79 (20%)	
				
*Stage*				0.16
I	160 (22%)	55 (18%)	105 (26%)	
II	214 (30%)	100 (32%)	114 (28%)	
III	204 (28%)	91 (29%)	113 (28%)	
IV	101 (14%)	45 (15%)	56 (14%)	
Unknown	39 (5.4%)	18 (5.8%)	21 (5.1%)	
				
*Tumour grade*				0.0009
Low	655 (92%)	269 (88%)	386 (95%)	
High	60 (8.4%)	38 (12%)	22 (5.4%)	
				
*Tumour border*				0.80
Expansile	543 (86%)	237 (86%)	306 (85%)	
Infiltrative	90 (14%)	38 (14%)	52 (15%)	
				
*p-MAPK3/1 expression*				
(−)	469 (70%)	227 (80%)	242 (63%)	<0.0001
(+)	202 (30%)	57 (20%)	145 (37%)	
				
*TP53 expression*				0.055
(−)	423 (59%)	194 (63%)	229 (56%)	
(+)	290 (41%)	112 (37%)	178 (44%)	
				
*FASN expression*				0.024
(−)	597 (84%)	267 (88%)	330 (81%)	
(+)	114 (16%)	38 (12%)	76 (19%)	
				
*MSI*				0.0021
MSI-low/MSS	591 (84%)	242 (79%)	349 (88%)	
MSI-high	113 (16%)	64 (21%)	49 (12%)	
				
*CIMP*				
CIMP-low/0	596 (85%)	237 (78%)	359 (89%)	<0.0001
CIMP-high	109 (15%)	66 (22%)	43 (11%)	
				
*LINE-1 methylation*				
⩾70%	121 (17%)	53 (18%)	68 (17%)	0.16
50–69%	497 (71%)	220 (74%)	277 (70%)	
<50%	79 (11%)	26 (8.7%)	53 (13%)	
				
*BRAF mutation*				0.048
(−)	602 (85%)	250 (82%)	352 (88%)	
(+)	104 (15%)	54(18%)	50 (12%)	
				
*KRAS mutation*				0.26
(−)	438 (62%)	195 (64%)	243 (60%)	
(+)	271 (38%)	109 (36%)	162 (40%)	
				
*PIK3CA* mutation				0.79
(−)	538 (84%)	236 (84%)	302 (83%)	
(+)	106 (16%)	45 (16%)	61 (17%)	

Abbreviations: BMI=body mass index; CIMP=CpG island methylator phenotype; FASN=fatty acid synthase; MSI=microsatellite instability; MSS=microsatellite stable; p-AMPK=phosphorylated AMP-activated protein kinase; p-MAPK3/1=phosphorylated mitogen-activated protein kinase.

% Number indicated the proportion of cases with a given clinical, pathologic or molecular feature among all cases, p-AMPK-negative cases or p-AMPK-positive cases.

**Table 2:** p-AMPK status in colorectal cancer and patient mortality

		**Colorectal cancer-specific mortality**			**Overall mortality**		
**AMPK status**	**Total *N***	**Deaths/ person-years**	**Univariate HR (95% CI)**	**Multivariate stage-matched HR (95% CI)**	**Deaths/ person-years**	**Univariate HR (95% CI)**	**Multivariate stage-matched HR (95% CI)**
p-AMPK (−)	309	86/2164	1 (referent)	1 (referent)	125/2164	1 (referent)	1 (referent)
p-AMPK (+)	409	108/2952	0.84 (0.61–1.17)	0.95 (0.71–1.28)	181/2952	1.08 (0.84–1.39)	1.12 (0.89–1.42)

Abbreviations: BMI=body mass index; CI=confidence interval; HR=hazard ratio; CIMP=CpG island methylator phenotype; FASN=fatty acid synthase; MSI=microsatellite instability; p-AMPK=phosphorylated AMP-activated protein kinase.

The multivariate, stage-matched (stratified) Cox model initially included sex, age at diagnosis, year of diagnosis, BMI, family history of colorectal cancer, tumour location, tumour grade, tumour border, CIMP, MSI, LINE-1 methylation, *BRAF*, *KRAS*, *PIK3CA*, TP53 and FASN. A backward stepwise elimination with a threshold of *P*=0.20 was used to select variables in the final model. Stage adjustment (I, IIA, IIB, IIIA, IIIB, IIIC, IV, unknown) was done using the ‘strata’ option in the SAS ‘proc phreg’ command.

**Table 3:** p-AMPK status and patient mortality in strata of p-MAPK3/1 status (upper rows) and p-MAPK3/1 status and patient mortality in strata of p-AMPK status (lower rows)

	**Colorectal cancer-specific mortality**			**Overall mortality**		
	**No. of deaths/cases**	**Univariate HR (95% CI)**	**Multivariate stage-matched HR (95% CI)**	**No. of deaths/cases**	**Univariate HR (95% CI)**	**Multivariate stage-matched HR (95% CI)**
*p-MAPK3/1* (−)						
p-AMPK (−)	59/227	1 (referent)	1 (referent)	84/227	1 (referent)	1 (referent)
p-AMPK (+)	72/242	1.14 (0.81–1.61)	1.22 (0.85–1.75)	106/242	1.20 (0.90–1.60)	1.31 (0.98–1.76)
						
*p-MAPK3/1* (+)						
p-AMPK (−)	23/57	1 (referent)	1 (referent)	32/57	1 (referent)	1 (referent)
p-AMPK (+)	27/145	0.39 (0.23–0.69)	0.42 (0.24–0.74)	62/145	0.64 (0.42–0.98)	0.65 (0.42–1.01)
						
*p-AMPK* (−)						
p-MAPK3/1 (−)	59/227	1 (referent)	1 (referent)	84/227	1 (referent)	1 (referent)
p-MAPK3/1 (+)	23/57	1.75 (1.08–2.82)	1.94 (1.17–3.24)	32/57	1.67 (1.12–2.50)	1.88 (1.23–2.86)
						
*p-AMPK* (+)						
p-MAPK3/1 (−)	72/242	1 (referent)	1 (referent)	106/242	1 (referent)	1 (referent)
p-MAPK3/1 (+)	27/145	0.55 (0.36–0.85)	0.55 (0.35–0.86)	62/145	0.84 (0.62–1.14)	0.80 (0.58–1.10)
*P*_interaction_ (p-AMPK and p-MAPK3/1)	0.0014	0.0017		0.016	0.0026	

Abbreviations: BMI=body mass index; CI=confidence interval; HR=hazard ratio; p-AMPK=phosphorylated AMP-activated protein kinase; p-MAPK3/1=phosphorylated mitogen-activated protein kinase. The multivariate, stage-matched (stratified) Cox model included p-AMPK variable stratified by p-MAPK3/1 status (or p-MAPK3/1 variable stratified by p-AMPK status), sex, age, year of diagnosis, BMI, tumour location, tumour grade, tumour border, CIMP, MSI, LINE-1 methylation, *BRAF*, *KRAS*, *PIK3CA*, TP53 and FASN. A backward stepwise elimination with a threshold of *P*=0.20 was used to select variables in the final model. Stage adjustment (I, IIA, IIB, IIIA, IIIB, IIIC, IV, unknown) was done using the ‘strata’ option in the SAS ‘proc phreg’ command.

## References

[bib1] Beeram M, Patnaik A, Rowinsky EK (2005) Raf: a strategic target for therapeutic development against cancer. J Clin Oncol 23: 6771–6790 16170185 10.1200/JCO.2005.08.036

[bib2] Boland CR, Thibodeau SN, Hamilton SR, Sidransky D, Eshleman JR, Burt RW, Meltzer SJ, Rodriguez-Bigas MA, Fodde R, Ranzani GN, Srivastava S (1998) A National Cancer Institute Workshop on Microsatellite Instability for cancer detection and familial predisposition: development of international criteria for the determination of microsatellite instability in colorectal cancer. Cancer Res 58: 5248–5257 9823339

[bib3] Bowker SL, Majumdar SR, Veugelers P, Johnson JA (2006) Increased cancer-related mortality for patients with type 2 diabetes who use sulfonylureas or insulin. Diabetes Care 29: 254–258 16443869 10.2337/diacare.29.02.06.dc05-1558

[bib4] Buzzai M, Jones RG, Amaravadi RK, Lum JJ, DeBerardinis RJ, Zhao F, Viollet B, Thompson CB (2007) Systemic treatment with the antidiabetic drug metformin selectively impairs p53-deficient tumor cell growth. Cancer Res 67: 6745–6752 17638885 10.1158/0008-5472.CAN-06-4447

[bib5] Chan AT, Ogino S, Fuchs CS (2007) Aspirin and the risk of colorectal cancer in relation to the expression of COX-2. N Engl J Med 356: 2131–2142 17522398 10.1056/NEJMoa067208

[bib6] Contreras CM, Gurumurthy S, Haynie JM, Shirley LJ, Akbay EA, Wingo SN, Schorge JO, Broaddus RR, Wong KK, Bardeesy N, Castrillon DH (2008) Loss of Lkb1 provokes highly invasive endometrial adenocarcinomas. Cancer Res 68: 759–766 18245476 10.1158/0008-5472.CAN-07-5014

[bib7] Esteve-Puig R, Canals F, Colome N, Merlino G, Recio JA (2009) Uncoupling of the LKB1-AMPKalpha energy sensor pathway by growth factors and oncogenic BRAF. PLoS ONE 4: e4771 19274086 10.1371/journal.pone.0004771 PMC2651576

[bib8] Evans JM, Donnelly LA, Emslie-Smith AM, Alessi DR, Morris AD (2005) Metformin and reduced risk of cancer in diabetic patients. Bmj 330: 1304–1305 15849206 10.1136/bmj.38415.708634.F7 PMC558205

[bib9] Fay JR, Steele V, Crowell JA (2009) Energy homeostasis and cancer prevention: the AMP-activated protein kinase. Cancer Prev Res (Philadelphia, PA) 2: 301–309 10.1158/1940-6207.CAPR-08-0166 19336731

[bib10] Fluge O, Gravdal K, Carlsen E, Vonen B, Kjellevold K, Refsum S, Lilleng R, Eide TJ, Halvorsen TB, Tveit KM, Otte AP, Akslen LA, Dahl O (2009) Expression of EZH2 and Ki-67 in colorectal cancer and associations with treatment response and prognosis. Br J Cancer 101: 1282–1289 19773751 10.1038/sj.bjc.6605333 PMC2768450

[bib11] Gaber A, Johansson M, Stenman UH, Hotakainen K, Ponten F, Glimelius B, Bjartell A, Jirstrom K, Birgisson H (2009) High expression of tumour-associated trypsin inhibitor correlates with liver metastasis and poor prognosis in colorectal cancer. Br J Cancer 100: 1540–1548 19384300 10.1038/sj.bjc.6605047 PMC2696764

[bib12] Hadad SM, Baker L, Quinlan PR, Robertson KE, Bray SE, Thomson G, Kellock D, Jordan LB, Purdie CA, Hardie DG, Fleming S, Thompson AM (2009) Histological evaluation of AMPK signalling in primary breast cancer. BMC Cancer 9: 307 19723334 10.1186/1471-2407-9-307 PMC2744705

[bib13] Hughes LA, van den Brandt PA, de Bruine AP, Wouters KA, Hulsmans S, Spiertz A, Goldbohm RA, de Goeij AF, Herman JG, Weijenberg MP, van Engeland M (2009) Early life exposure to famine and colorectal cancer risk: a role for epigenetic mechanisms. PLoS One 4: e7951 19956740 10.1371/journal.pone.0007951 PMC2776970

[bib14] Hwang JT, Kim YM, Surh YJ, Baik HW, Lee SK, Ha J, Park OJ (2006) Selenium regulates cyclooxygenase-2 and extracellular signal-regulated kinase signaling pathways by activating AMP-activated protein kinase in colon cancer cells. Cancer Res 66: 10057–10063 17047069 10.1158/0008-5472.CAN-06-1814

[bib15] Inoki K, Guan KL (2009) Tuberous sclerosis complex, implication from a rare genetic disease to common cancer treatment. Hum Mol Genet 18: R94–100 19297407 10.1093/hmg/ddp032 PMC2657945

[bib16] Irahara N, Nosho K, Baba Y, Shima K, Lindeman NI, Hazra A, Schernhammer ES, Hunter DJ, Fuchs CS, Ogino S (2010) Precision of pyrosequencing assay to measure LINE-1 methylation in colon cancer, normal colonic mucosa, and peripheral blood cells. J Mol Diagn 12: 177–183 20093385 10.2353/jmoldx.2010.090106 PMC2871724

[bib17] Jemal A, Siegel R, Ward E, Hao Y, Xu J, Thun MJ (2009) Cancer statistics, 2009. CA Cancer J Clin 59: 225–249 19474385 10.3322/caac.20006

[bib18] Ji H, Ramsey MR, Hayes DN, Fan C, McNamara K, Kozlowski P, Torrice C, Wu MC, Shimamura T, Perera SA, Liang MC, Cai D, Naumov GN, Bao L, Contreras CM, Li D, Chen L, Krishnamurthy J, Koivunen J, Chirieac LR, Padera RF, Bronson RT, Lindeman NI, Christiani DC, Lin X, Shapiro GI, Janne PA, Johnson BE, Meyerson M, Kwiatkowski DJ, Castrillon DH, Bardeesy N, Sharpless NE, Wong KK (2007) LKB1 modulates lung cancer differentiation and metastasis. Nature 448: 807–810 17676035 10.1038/nature06030

[bib19] Jubb AM, Turley H, Moeller HC, Steers G, Han C, Li JL, Leek R, Tan EY, Singh B, Mortensen NJ, Noguera-Troise I, Pezzella F, Gatter KC, Thurston G, Fox SB, Harris AL (2009) Expression of delta-like ligand 4 (Dll4) and markers of hypoxia in colon cancer. Br J Cancer 101: 1749–1757 19844231 10.1038/sj.bjc.6605368 PMC2778546

[bib20] Kim HS, Hwang JT, Yun H, Chi SG, Lee SJ, Kang I, Yoon KS, Choe WJ, Kim SS, Ha J (2008) Inhibition of AMP-activated protein kinase sensitizes cancer cells to cisplatin-induced apoptosis via hyper-induction of p53. J Biol Chem 283: 3731–3742 18079115 10.1074/jbc.M704432200

[bib21] Kim J, Yoon MY, Choi SL, Kang I, Kim SS, Kim YS, Choi YK, Ha J (2001) Effects of stimulation of AMP-activated protein kinase on insulin-like growth factor 1- and epidermal growth factor-dependent extracellular signal-regulated kinase pathway. J Biol Chem 276: 19102–19110 11262401 10.1074/jbc.M011579200

[bib22] Kim MJ, Park IJ, Yun H, Kang I, Choe W, Kim SS, Ha J (2010) AMP-activated protein kinase antagonizes pro-apoptotic extracellular signal-regulated kinase activation by inducing dual-specificity protein phosphatases in response to glucose deprivation in HCT116 carcinoma. J Biol Chem 285(19): 14617–14627 20220132 10.1074/jbc.M109.085456 PMC2863197

[bib23] Kontos CK, Papadopoulos IN, Fragoulis EG, Scorilas A (2010) Quantitative expression analysis and prognostic significance of L-DOPA decarboxylase in colorectal adenocarcinoma. Br J Cancer 102: 1384–1390 20424616 10.1038/sj.bjc.6605654 PMC2865762

[bib24] Loupakis F, Ruzzo A, Cremolini C, Vincenzi B, Salvatore L, Santini D, Masi G, Stasi I, Canestrari E, Rulli E, Floriani I, Bencardino K, Galluccio N, Catalano V, Tonini G, Magnani M, Fontanini G, Basolo F, Falcone A, Graziano F (2009) KRAS codon 61, 146 and BRAF mutations predict resistance to cetuximab plus irinotecan in KRAS codon 12 and 13 wild-type metastatic colorectal cancer. Br J Cancer 101: 715–721 19603018 10.1038/sj.bjc.6605177 PMC2736831

[bib25] Ma L, Chen Z, Erdjument-Bromage H, Tempst P, Pandolfi PP (2005) Phosphorylation and functional inactivation of TSC2 by Erk implications for tuberous sclerosis and cancer pathogenesis. Cell 121: 179–193 15851026 10.1016/j.cell.2005.02.031

[bib26] Milde-Langosch K, Bamberger AM, Rieck G, Grund D, Hemminger G, Muller V, Loning T (2005) Expression and prognostic relevance of activated extracellular-regulated kinases (ERK1/2) in breast cancer. Br J Cancer 92: 2206–2215 15928662 10.1038/sj.bjc.6602655 PMC2361826

[bib27] Nosho K, Irahara N, Shima K, Kure S, Kirkner GJ, Schernhammer ES, Hazra A, Hunter DJ, Quackenbush J, Spiegelman D, Giovannucci EL, Fuchs CS, Ogino S (2008a) Comprehensive biostatistical analysis of CpG island methylator phenotype in colorectal cancer using a large population-based sample. PLoS ONE 3: e3698 19002263 10.1371/journal.pone.0003698 PMC2579485

[bib28] Nosho K, Kawasaki T, Ohnishi M, Suemoto Y, Kirkner GJ, Zepf D, Yan L, Longtine JA, Fuchs CS, Ogino S (2008b) PIK3CA mutation in colorectal cancer: relationship with genetic and epigenetic alterations. Neoplasia 10: 534–541 18516290 10.1593/neo.08336 PMC2386538

[bib29] Nosho K, Shima K, Irahara N, Kure S, Firestein R, Baba Y, Toyoda S, Chen L, Hazra A, Giovannucci EL, Fuchs CS, Ogino S (2009) SIRT1 histone deacetylase expression is associated with microsatellite instability and CpG island methylator phenotype in colorectal cancer. Mod Pathol 22: 922–932 19430421 10.1038/modpathol.2009.49 PMC2704253

[bib30] Ogino S, Brahmandam M, Cantor M, Namgyal C, Kawasaki T, Kirkner G, Meyerhardt JA, Loda M, Fuchs CS (2006a) Distinct molecular features of colorectal carcinoma with signet ring cell component and colorectal carcinoma with mucinous component. Mod Pathol 19: 59–68 16118624 10.1038/modpathol.3800482

[bib31] Ogino S, Brahmandam M, Kawasaki T, Kirkner GJ, Loda M, Fuchs CS (2006b) Combined analysis of COX-2 and p53 expressions reveals synergistic inverse correlations with microsatellite instability and CpG island methylator phenotype in colorectal cancer. Neoplasia 8: 458–464 16820091 10.1593/neo.06247 PMC1601473

[bib32] Ogino S, Cantor M, Kawasaki T, Brahmandam M, Kirkner GJ, Weisenberger DJ, Campan M, Laird PW, Loda M, Fuchs CS (2006c) CpG island methylator phenotype (CIMP) of colorectal cancer is best characterised by quantitative DNA methylation analysis and prospective cohort studies. Gut 55: 1000–1006 16407376 10.1136/gut.2005.082933 PMC1856352

[bib33] Ogino S, Goel A (2008) Molecular classification and correlates in colorectal cancer. J Mol Diagn 10: 13–27 18165277 10.2353/jmoldx.2008.070082 PMC2175539

[bib34] Ogino S, Kawasaki T, Brahmandam M, Yan L, Cantor M, Namgyal C, Mino-Kenudson M, Lauwers GY, Loda M, Fuchs CS (2005) Sensitive sequencing method for KRAS mutation detection by pyrosequencing. J Mol Diagn 7: 413–421 16049314 10.1016/S1525-1578(10)60571-5 PMC1867544

[bib35] Ogino S, Kawasaki T, Kirkner GJ, Kraft P, Loda M, Fuchs CS (2007) Evaluation of markers for CpG island methylator phenotype (CIMP) in colorectal cancer by a large population-based sample. J Mol Diagn 9: 305–314 17591929 10.2353/jmoldx.2007.060170 PMC1899428

[bib36] Ogino S, Kawasaki T, Kirkner GJ, Loda M, Fuchs CS (2006d) CpG island methylator phenotype-low (CIMP-low) in colorectal cancer: possible associations with male sex and KRAS mutations. J Mol Diagn 8: 582–588 17065427 10.2353/jmoldx.2006.060082 PMC1876166

[bib37] Ogino S, Kawasaki T, Nosho K, Ohnishi M, Suemoto Y, Kirkner GJ, Fuchs CS (2008a) LINE-1 hypomethylation is inversely associated with microsatellite instability and CpG island methylator phenotype in colorectal cancer. Int J Cancer 122: 2767–2773 18366060 10.1002/ijc.23470 PMC2630175

[bib38] Ogino S, Nosho K, Kirkner GJ, Kawasaki T, Chan AT, Schernhammer ES, Giovannucci EL, Fuchs CS (2008b) A cohort study of tumoral LINE-1 hypomethylation and prognosis in colon cancer. J Natl Cancer Inst 100: 1734–1738 19033568 10.1093/jnci/djn359 PMC2639290

[bib39] Ogino S, Nosho K, Kirkner GJ, Kawasaki T, Meyerhardt JA, Loda M, Giovannucci EL, Fuchs CS (2009) CpG island methylator phenotype, microsatellite instability, BRAF mutation and clinical outcome in colon cancer. Gut 58: 90–96 18832519 10.1136/gut.2008.155473 PMC2679586

[bib40] Ogino S, Nosho K, Meyerhardt JA, Kirkner GJ, Chan AT, Kawasaki T, Giovannucci EL, Loda M, Fuchs CS (2008c) Cohort study of fatty acid synthase expression and patient survival in colon cancer. J Clin Oncol 26: 5713–5720 18955444 10.1200/JCO.2008.18.2675 PMC2630484

[bib41] Ogino S, Odze RD, Kawasaki T, Brahmandam M, Kirkner GJ, Laird PW, Loda M, Fuchs CS (2006e) Correlation of pathologic features with CpG island methylator phenotype (CIMP) by quantitative DNA methylation analysis in colorectal carcinoma. Am J Surg Pathol 30: 1175–1183 16931963 10.1097/01.pas.0000213266.84725.d0

[bib42] Pelloski CE, Lin E, Zhang L, Yung WK, Colman H, Liu JL, Woo SY, Heimberger AB, Suki D, Prados M, Chang S, Barker III FG, Fuller GN, Aldape KD (2006) Prognostic associations of activated mitogen-activated protein kinase and Akt pathways in glioblastoma. Clin Cancer Res 12: 3935–3941 16818690 10.1158/1078-0432.CCR-05-2202

[bib43] Pollak M (2008) Insulin and insulin-like growth factor signalling in neoplasia. Nat Rev Cancer 8: 915–928 19029956 10.1038/nrc2536

[bib44] Rasheed S, Harris AL, Tekkis PP, Turley H, Silver A, McDonald PJ, Talbot IC, Glynne-Jones R, Northover JM, Guenther T (2009) Hypoxia-inducible factor-1alpha and -2alpha are expressed in most rectal cancers but only hypoxia-inducible factor-1alpha is associated with prognosis. Br J Cancer 100: 1666–1673 19436307 10.1038/sj.bjc.6605026 PMC2696753

[bib45] Rego RL, Foster NR, Smyrk TC, Le M, O’Connell MJ, Sargent DJ, Windschitl H, Sinicrope FA (2010) Prognostic effect of activated EGFR expression in human colon carcinomas: comparison with EGFR status. Br J Cancer 102: 165–172 19997103 10.1038/sj.bjc.6605473 PMC2813748

[bib46] Roth AD, Tejpar S, Delorenzi M, Yan P, Fiocca R, Klingbiel D, Dietrich D, Biesmans B, Bodoky G, Barone C, Aranda E, Nordlinger B, Cisar L, Labianca R, Cunningham D, Van Cutsem E, Bosman F (2010) Prognostic role of KRAS and BRAF in stage II and III resected colon cancer: results of the translational study on the PETACC-3, EORTC 40993, SAKK 60–00 trial. J Clin Oncol 28: 466–474 20008640 10.1200/JCO.2009.23.3452

[bib47] Schmitz KJ, Wohlschlaeger J, Alakus H, Bohr J, Stauder MA, Worm K, Winde G, Schmid KW, Baba HA (2007) Activation of extracellular regulated kinases (ERK1/2) but not AKT predicts poor prognosis in colorectal carcinoma and is associated with k-ras mutations. Virchows Arch 450: 151–159 17149612 10.1007/s00428-006-0342-y

[bib48] Schubbert S, Shannon K, Bollag G (2007) Hyperactive Ras in developmental disorders and cancer. Nat Rev Cancer 7: 295–308 17384584 10.1038/nrc2109

[bib49] Shackelford DB, Shaw RJ (2009) The LKB1-AMPK pathway: metabolism and growth control in tumour suppression. Nat Rev Cancer 9: 563–575 19629071 10.1038/nrc2676 PMC2756045

[bib50] Slattery ML, Curtin K, Wolff RK, Herrick JS, Caan BJ, Samowitz W (2010) Diet, physical activity, and body size associations with rectal tumor mutations and epigenetic changes. Cancer Causes Contr 21(8): 1237–1245 10.1007/s10552-010-9551-4 PMC2904420 20383576

[bib51] Vazquez-Martin A, Lopez-Bonet E, Oliveras-Ferraros C, Perez-Martinez MC, Bernado L, Menendez JA (2009) Mitotic kinase dynamics of the active form of AMPK (phospho-AMPKalphaThr172) in human cancer cells. Cell Cycle 8: 788–791 19221486 10.4161/cc.8.5.7787

[bib52] Weisenberger DJ, Siegmund KD, Campan M, Young J, Long TI, Faasse MA, Kang GH, Widschwendter M, Weener D, Buchanan D, Koh H, Simms L, Barker M, Leggett B, Levine J, Kim M, French AJ, Thibodeau SN, Jass J, Haile R, Laird PW (2006) CpG island methylator phenotype underlies sporadic microsatellite instability and is tightly associated with BRAF mutation in colorectal cancer. Nat Genet 38: 787–793 16804544 10.1038/ng1834

[bib53] Yeh JJ, Routh ED, Rubinas T, Peacock J, Martin TD, Shen XJ, Sandler RS, Kim HJ, Keku TO, Der CJ (2009) KRAS/BRAF mutation status and ERK1/2 activation as biomarkers for MEK1/2 inhibitor therapy in colorectal cancer. Mol Cancer Ther 8: 834–843 19372556 10.1158/1535-7163.MCT-08-0972 PMC2729756

[bib54] Zakikhani M, Dowling RJ, Sonenberg N, Pollak MN (2008) The effects of adiponectin and metformin on prostate and colon neoplasia involve activation of AMP-activated protein kinase. Cancer Prev Res (Philadelphia, PA) 1: 369–375 10.1158/1940-6207.CAPR-08-0081 19138981

[bib55] Zheng B, Jeong JH, Asara JM, Yuan YY, Granter SR, Chin L, Cantley LC (2009) Oncogenic B-RAF negatively regulates the tumor suppressor LKB1 to promote melanoma cell proliferation. Mol Cell 33: 237–247 19187764 10.1016/j.molcel.2008.12.026 PMC2715556

[bib56] Zlobec I, Molinari F, Kovac M, Bihl MP, Altermatt HJ, Diebold J, Frick H, Germer M, Horcic M, Montani M, Singer G, Yurtsever H, Zettl A, Terracciano L, Mazzucchelli L, Saletti P, Frattini M, Heinimann K, Lugli A (2010) Prognostic and predictive value of TOPK stratified by KRAS and BRAF gene alterations in sporadic, hereditary and metastatic colorectal cancer patients. Br J Cancer 102: 151–161 19935791 10.1038/sj.bjc.6605452 PMC2813744

